# Bacteriophage FNU1 negates *Fusobacterium nucleatum* induced cell growth, migration and chemotherapy resistance in gastrointestinal cancer cells

**DOI:** 10.3389/fcimb.2025.1721411

**Published:** 2026-01-08

**Authors:** Mwila Kabwe, Birhanu Ayelign, Shoukat Afshar-Sterle, Michael Buchert, Joseph Tucci

**Affiliations:** 1 Department of Rural Clinical Sciences, La Trobe Rural Health School, La Trobe University, Bendigo, VIC, Australia; 2 Holsworth Biomedical Research Centre, La Trobe Rural Health School, La Trobe University, Bendigo, VIC, Australia; 3 La Trobe Institute for Molecular Science, La Trobe University, Bendigo, VIC, Australia; 4 Biomarker and Tuft Cell Laboratory, Olivia Newton-John Cancer Research Institute, Heidelberg, VIC, Australia; 5 School of Cancer Medicine, La Trobe University, Bundoora, VIC, Australia

**Keywords:** 5-fluorouracil resistance, apoptosis and necrosis, autophagy, cell migration, oxaliplatin resistance, phage therapy, gastric cancer, colon cancer

## Abstract

**Background:**

*Fusobacterium nucleatum* is an oncobacterium capable of promoting the growth and chemotherapy resistance of colonised tumours. Although *F. nucleatum* is usually susceptible to a range of antibiotics *in vitro*, these have been associated with worse outcomes when administered with anti-neoplastic chemotherapy. Bacteriophages are viewed as natural alternatives to antibiotics that provide bacterial-specific targeting.

**Methods:**

In this study, we have employed an *F. nucleatum* specific bacteriophage, FNU1, to limit the effects of this oncobacteria in colon cancer and gastric cancer cell models.

**Results:**

We demonstrated that FNU1 was able to negate the *F. nucleatum* induced growth stimulatory effects, migratory ability, autophagy, anti-apoptotic effects and chemotherapy resistance in these cell models.

**Conclusion:**

Treatments with bacteriophage FNU1, therefore, have the potential to augment existing cancer therapy, and further testing in animal models is warranted.

## 1 Introduction

Cancer is one of the leading causes of death worldwide (Naghavi et al., 2017). The gastrointestinal tract (GIT), which includes the oral cavity, pharynx, and other regions of the digestive tract, contains tissues and organs with the highest incidence of cancers, some of which are the leading cause of cancer-related deaths globally (Bray et al., 2018; Siegel et al., 2019). The GIT is a unique system eliciting innate and adaptive immune functions to keep out pathogens, as well as facilitating nutrient absorption (Wershil and Furuta, 2008). The symbiosis between the GIT and its microbiota is important in immune tolerance, immune modulation, and maintenance of metabolic homeostasis (Dey, 2019). The microbiome of the GIT is composed of bacteria and viruses, and dysbiosis in these tissues has been associated with changes in the metabolome, immune dysregulation, and cancer (Rajagopala et al., 2017; Hsu et al., 2019). Both bacteria and eukaryotic viruses have pathogenic mechanisms that have been linked directly to carcinogenesis (Rajagopala et al., 2017; Magalhaes et al., 2019). Bacterial viruses (bacteriophages) regulate bacterial populations leading to a dynamic (Hsu et al., 2019) and potentially manipulable microbiome. This characteristic of bacteriophages makes them an attractive resource for the potential manipulation of the microbiome in a dysbiotic state.

5-Fluorouracil (5-FU) and Oxaliplatin are part of a regime which is commonly used for both colon and gastric cancers (Masuishi et al., 2017; Wilson et al., 2024). 5-FU is phosphorylated to fluorodeoxyuridine triphosphate and fluorouridine triphosphate that incorporate into and cause damage to DNA and RNA, respectively. Oxaliplatin contains a reactive carbonium ion that forms intra-strand crosslinks in DNA, inhibiting the S phase of cell division (Mohelnikova-Duchonova et al., 2014). Treatment failure with both these antineoplastic agents is reported to be modulated by the tumour microbiome and is associated with recurrent disease (Sadeghloo and Sadeghi, 2025).

Genomic sequencing data have shown that a select few microbes are increased in tumour tissues (Kabwe et al., 2021a). Among several cancers, *Fusobacterium* is the most frequently increased and the most widely studied bacteria, especially in the context of colon cancers. *Fusobacterium nucleatum* is an heterogenous oral pathobiont (Zepeda-Rivera et al., 2025) known to promote tumourigenesis by dampening the host’s anti-tumour immunity, promoting the release of pro-inflammatory cytokines and directly stimulating the proliferation of tumour cells (Kabwe et al., 2021a). The interactions between *F. nucleatum* and colon cancer cells are complex. Studies have highlighted that, via the TLR4 receptor, *F. nucleatum* increases proliferation of colon cancer cells through inflammatory modulation (Yang et al., 2017), inhibits apoptosis by upregulation of BIRC3 [Baculoviral IAP (Inhibitor of Apoptosis Protein) Repeat-Containing 3] (Zhang et al., 2019), and modulates autophagy to promote chemotherapy resistance (Yu et al., 2017). It has been shown that the presence and overexpression of the sugar molecule Gal-GalNAc in colorectal cancer cells facilitates binding and cellular invasion of *F. nucleatum* through interaction with its protein *Fusobacterium* adhesin protein 2 (Fap2), which allows enrichment of *F. nucleatum* at tumour sites (Abed et al., 2016). Yet while the Gal-GalNAc molecule is widely expressed by several cancer tissues (Abed et al., 2017), the specific interaction between *F. nucleatum* and Gal-GalNAc has not been explored in gastric cancer.

The increased recognition of the role of *F. nucleatum* in promoting cancer growth has stimulated efforts to develop treatments for disrupting biofilms of oncobacteria in the tumour microenvironment. These treatments have the potential to augment and improve cancer therapy. The use of antibiotics to control *F. nucleatum* in colorectal cancer has been examined and, in some instances, found to improve chemotherapy outcomes in mice (Bullman et al., 2017). However, antibiotic treatment before chemotherapy has been associated with worse outcomes in some mouse models (Viaud et al., 2013) and in humans, where it is associated with disease progression and shortened survival (Routy et al., 2018; Ochi et al., 2021). Further, a history of antibiotic use, as people age, has been associated with an increased risk of development of GIT cancers (Wang et al., 2014; Boursi et al., 2015; Dik et al., 2016; Yan et al., 2019). Finally, the prevalence of *F. nucleatum* in tumourigenic tissues is independent of antibiotic use (Gaiser et al., 2019), indicating their minimal impact in controlling this oncobacterium. Recent JAVELIN and KEYNOTE-061 phase III international clinical trials have shown that GIT cancers do not respond to immunotherapy (Bang et al., 2018; Shitara et al., 2018; Yan et al., 2019), and evidence has suggested this may be a result of the human microbiota’s impact on gut inflammation and immune dysregulation (Meng et al., 2018). The potential for using bacteriophages against key oncobacteria in augmenting cancer therapies has been reviewed (Kabwe et al., 2021a), and bacteriophages have been applied to assist in the control of *F. nucleatum*-enhanced colorectal cancer (Zheng et al., 2019; Lam et al., 2025) in experimental animal models. However, extensive data on the specific role of bacteriophages in disrupting the oncobacterial-cancer cell nexus and enhancing chemotherapy efficacy in a range of GIT cancers, is lacking. This study aimed to investigate how the interaction between *F. nucleatum* and gastric tumour tissues is influenced by the *F. nucleatum*-specific bacteriophage FNU1. We explored the capacity of FNU1 to negate *F. nucleatum*-dependent growth stimulation, migration, autophagy, anti-apoptotic effects, and chemotherapy resistance in colon and gastric cancer cells.

## 2 Materials and methods

### 2.1 Ethics approval

All study protocols have been approved by the La Trobe University Ethics Committee (S17-112) and all methods performed in accordance with the La Trobe University Ethics, Biosafety and Integrity guidelines, and regulations.

### 2.2 Bacteria culture conditions and bacteriophage purification

The fully characterised *F. nucleatum* strain (ATCC10953) (Karpathy et al., 2007) and bacteriophage FNU1 (Accession number: MK554696) were used to elucidate the interactions between bacteria, bacteriophage, and cancer cells. The bacteria were cultured in Brain Heart Infusion (BHI; Oxoid™, Australia) media supplemented with 0.5% (w/v) cysteine (Sigma-Aldrich^®^, Australia) and 0.5% (v/v) haemin (Sigma-Aldrich^®^, Australia). The bacterial cultures were incubated at 37°C under anaerobic conditions using AnaeroGen pack (Oxoid™, Australia) (Kabwe et al., 2019).

The broth macrodilution method (Wiegand et al., 2008) was used to determine the minimum inhibitory concentration of ampicillin against *F. nucleatum*. Ampicillin was serially diluted (by 1 in 2 serial dilutions from 10.0 mcg/mL) in BHI broth containing 5×10^5^ CFU/mL. The dilutions were then plated onto BHI agar plates and incubated for 48 h anaerobically at 37 °C before performing colony counts.

Bacteriophage FNU1 was clonally expanded from one plaque, then subjected to 0.2 µm filtration. This was termed the “crude” stock, as it was not “purified” of lipopolysaccharides (LPS) and endotoxin. For purification, this crude stock was precipitated in sodium chloride (NaCl) and polyethylene-glycol (PEG 8000) (Sigma-Aldrich^®^, Australia) and treated with 2% (v/v) Triton™ X-100 (Sigma-Aldrich^®^, Australia) (Branston et al., 2015) before washing three times in phosphate-buffered saline (PBS; pH 7.4) and resuspending in 10% (v/v) FBS supplemented RPMI-1640 medium containing L-glutamine and sodium bicarbonate (Sigma-Aldrich^®^, Australia) (Kabwe et al., 2021b). Bacteriophages were added to the *F. nucleatum*/cancer cell co-culture at a multiplicity of infection (MOI) of 0.1 (with reference to *F. nucleatum* cells).

### 2.3 Human cell line maintenance

Colon HCT116 cells (ATCC, USA) and gastric MKN1 (ATCC, USA) cancer cell lines purchased from the American Type Culture Collection were maintained in 10% (v/v) fetal bovine serum (FBS; Sigma-Aldrich^®^, Australia) supplemented RPMI-1640 medium containing L-glutamine and sodium bicarbonate (Sigma-Aldrich^®^, Australia). Cells were passaged by detachment with 1× trypsin-EDTA solution (0.05% trypsin, 0.02% EDTA, in Hanks′ Balanced Salt Solution (Sigma-Aldrich^®^) and maintained in humidified 5% CO_2_ at 37 °C. Colon and gastric cancer cell lines were grown up to 90% confluence before harvesting with 1× trypsin and resuspending in fresh 10% (v/v) FBS-supplemented RPMI-1640 medium containing L-glutamine and sodium bicarbonate (Sigma-Aldrich^®^, Australia) to a concentration of 1×10^4^ cells per mL.

### 2.4 Bacterial/bacteriophage and cancer cells co-cultures

Colon and gastric cancer cell lines were grown up to 90% confluence before harvesting with 1× trypsin-EDTA and resuspending in fresh 10% (v/v) FBS-supplemented RPMI-1640 medium containing L-glutamine and sodium bicarbonate (Sigma-Aldrich^®^, Australia) to a concentration of 1×10^4^ cells per mL.

Exponentially growing bacteria in BHI broth were harvested to a concentration of 1×10^8^ colony forming units (CFU) per mL. One mL containing 1×10^8^ cells of *F. nucleatum* was washed 3 times by centrifugation at 12, 000 × ℊ and resuspended in RPMI-1640 medium containing L-glutamine and sodium bicarbonate (Sigma-Aldrich^®^, Australia). *F. nucleatum* in RPMI-1640 was then added to the 1×10^4^ cells per mL of cancer cells at an MOI of 10 (throughout the manuscript, this combination is termed the co-culture).

*F. nucleatum*/cancer cell co-cultures treated with FNU1 were incubated in humidified 5% CO_2_ at 37 °C for 24 h. The *F. nucleatum* infection was confirmed by passaging the adherent cells from co-culture using trypsin-EDTA as described above in section 2.3, except that the infected cells were resuspended in an equal volume of fresh BHI broth and then inoculated onto BHI agar to observe colonies. Identity of the bacteria was confirmed by PCR using 16s rRNA primers (U27F: 5’ - AGAGTTTGATCMTGGCTCAG – 3’ and U1492R: 5’ - AAGGAGGTGWTCCARCC - 3’) under thermocycling conditions: 95 °C for 3 min; 32 cycles of 95 °C for 30 s, 60 °C for 30 s, and 72 °C for 90 s; then a final extension at 72 °C for 10 min (Kabwe et al., 2019) and *F. nucleatum* specific primers (5’ – AGTTGTCCTATACCAGCTCCAAC - 3’ and 5’ – GCAACATTCTTTGCAGCACGTACTGC – 3’) under thermocycling conditions: 95 °C for 10 min; 35 cycles of 95 °C for 30 s, 60 °C for 30 s, and 72 °C for 45 s; then a final extension at 72 °C for 10 min.

### 2.5 Visualisation of Gal-GalNAc using immunofluorescence microscopy

Human gastric MKN1 cells and colon HCT116 tumour cells at 1×10^4^ cells per mL were seeded in 24-well plates and after overnight culture, the cells were washed three times with RPMI 1640 cell growth medium and fixed with 4% paraformaldehyde. Cells were blocked with 1% Bovine Serum Albumin (BSA) for 30 min at room temperature. Fluorescein isothiocyanate conjugated lectin (FITC-lectin, specific for the sugar molecule D-galactose-β (1–3)-N-acetyl-D-galactosamine (Gal-GalNAc), ThermoFisher Scientific, Australia) was then incubated (50 μg/mL in PBS) overnight at 4°C, followed by staining with Hoechst 33342 (1:1000 in PBS) for 30 min at room temperature. Finally, the slides were mounted with Fluoromount-G™ Mounting Medium (Invitrogen, ThermoFisher Scientific, Australia). Images were visualised using Zeiss LSM 980 Axio Observer 7 inverted confocal microscope.

### 2.6 Visualisation of Gal-GalNAc by immunohistochemistry

Mouse gastric tumour tissues were provided by Dr Moritz Eissmann, Cancer and Inflammatory Laboratory, Olivia Newton Cancer Research Institute, Heidelberg, Australia. The slides were deparaffinised using xylene for 15 min twice, 100% alcohol twice and 70% alcohol for 5 min, then treated with antigen retrieval solution (10mM of sodium citrate pH 6) in a microwave for 20 min and then left for 40 min at room temperature, followed by a final wash for 5 min in PBS to remove any impurities. Non-specific binding was minimised by blocking the slides with a solution of PBS supplemented with 10% FBS, 10% BSA, and 5% Triton-X for one hour at room temperature. Slides were then incubated overnight at 4 °C in 50 μg/mL in PBS of FITC-conjugated lectin followed by incubation with Hoechst 33342 (1:1000 in PBS) for 30 min at room temperature. Finally, the slides were mounted with Fluoromount-G™ Mounting Medium (Invitrogen, ThermoFisher Scientific, Australia) and visualised using an immunofluorescence microscope.

### 2.7 Quantification of Gal-GalNAc expression by flow cytometry analysis

The expression level of Gal-GalNAc in *Kras^G12D^*, *Pi3kCA^H1047R^* and *Tp53^R172H^* (KPT) mutant murine gastric cancer organoids and human gastrointestinal cancer cells was measured using flow cytometry. The KPT organoids were generated and maintained as previously (Huber et al., 2024; Konecnik et al., 2025). To obtain single cells, murine cancer organoids were dissociated from the Matrigel using 1× trypsin with EDTA (0.05% trypsin, 0.02% EDTA). Briefly, cells were enzymatically detached with 1× trypsin with EDTA and cells were washed three times with RPMI medium. Cells were blocked with 1% BSA for 30 min at room temperature. Single cells were stained with FITC-conjugated lectin at a concentration of 50 µg/mL in PBS for 30 min at room temperature and then washed three times in PBS. The cells were stained with Sytox blue before being analysed by flow cytometry. The data was analysed using FlowJo 10.10 software.

### 2.8 Laser scanning confocal microscopy and *F. nucleatum* invasion

Exponentially growing *F. nucleatum* was adjusted to 1×10^8^ CFU/mL in PBS (pH 7.4) and stained with the CellTrace™ CFSE cell proliferation reagent according to manufacturer instructions (Invitrogen™, Australia). Briefly, 2 µl/mL were added to the *F. nucleatum* suspension and incubated for 30 min. CellTrace™ CFSE-stained *F. nucleatum* were resuspended in fresh 10% (v/v) FBS-supplemented RPMI-1640 medium containing L-glutamine and sodium bicarbonate (Sigma-Aldrich^®^, Australia) and added to 1×10^4^ cells per mL of cancer cells at an MOI of 10. The co-culture was then transferred to 6-well plates with coverslips and incubated in humidified 5% CO_2_ at 37 °C for 24 h. Unattached bacteria were washed off in fresh RPMI-1640 media twice before adding fresh media containing bacteriophage FNU1 (MOI = 0.1 to the initial starting concentration of *F. nucleatum*) or ampicillin (1mg/mL; Sigma-Aldrich^®^, Australia) and incubating for a further 2 h in humidified 5% CO_2_ at 37 °C. The RPMI-1640 media was then removed, and excess media washed off three times in PBS before staining the cell nucleus with Hoechst 33342 (2 µl/mL; Sigma-Aldrich^®^, Australia) and cytoplasm with CellBrite^®^ Red cytoplasmic membrane dye (2 µl/mL; Biotium, Australia). The cells were then incubated for 30 min before washing off in fresh RPMI-1640 media and incubating for a further 5 min at 37 °C. The RPMI medium was then washed off in PBS and the cover slips were mounted on microscope slides with PBS, sealed with nail polish and examined immediately under the Olympus Fluoview Fv10i confocal laser scanning microscope (Olympus Life Science, Australia). The three-channel excitation and emission (Ex/Em) wavelengths were 492/517 nm (CellTrace™ CFSE), 644/665 nm (CellBrite^®^ Red) and 361/497 nm (Hoechst 33342). Images were processed in ImageJ 1.54p and visualised using a false-colour palette to highlight the different components of the cell–bacteria co-culture.

### 2.9 Cell proliferation assay

Cell proliferation was quantified using the Sulforhodamine B (SRB) assay (Orellana and Kasinski, 2016). The SRB assay widely used for cytotoxic assessment has been evaluated against other metabolic assays and measures total cellular biomass without being influenced by chemicals that may alter cellular activity directly or indirectly (Bauer et al., 2025). Co-cultures treated with 5 -FU (20 µM) and oxaliplatin (20 µM) were incubated in humidified 5% CO_2_ at 37 °C for 24 h before fixation in 10% (w/v) trichloroacetic acid (ThermoFisher Scientific, Australia) at 4 °C for 30 min. The trichloroacetic acid was gently pipetted off, and fixed cells were washed in milli-Q water (Merck Milli-Q water system, Australia) five times and air dried at room temperature. The cells were then stained with 1% (w/v) SRB (Sigma-Aldrich^®^, Australia) in glacial acetic acid (ThermoFisher Scientific, Australia) for 15 min. The SRB was then pipetted off and excess stain washed off in 1% (v/v) glacial acetic acid and air-dried. SRB that remained bound to the cells was solubilised in 10 mM unbuffered TRIS base and its absorbance at 540 nm quantified using the FlexStation 3 plate reader (Molecular Devices, United States).

### 2.10 Wound healing migration assay

To determine the effect of *F. nucleatum* infection and FNU1 bacteriophage treatment on migration of MKN1 gastric cancer cells and HCT116 colon cancer cells, we performed wound healing or scratch assays under different conditions: (i) uninfected cells, (ii) uninfected cells treated with FNU1, (iii) *F. nucleatum* infected GI cancer cells, and (iv) *F. nucleatum* infected cells treated with FNU1. Migration was quantified by measuring the percentage of wound closure at 24-, 48- and 72-h post-wound scratch. Wound healing assays were performed according to the protocol used by Ou and colleagues (Ou et al., 2023). In brief, 1 × 10^5^ MKN1 cells and HCT116 cells were seeded in 6-well plates and incubated at 37 °C and grown to 100% confluence. The cells of each well were then infected with *F. nucleatum* at 1:30 MOI, or an equal volume of PBS as a control, and incubated for 2 h. The cell monolayers were then scratched with a 20 µl pipette tip in a straight line to create a “wound” followed by two washes with PBS and incubation for 24 h by adding serum-free medium and 1:10 MOI of FNU bacteriophage. Images of the same scratched area were taken using a ZeissAxio observer widefield microscope at 0, 24, 48 and 72 h. The scratched area was measured by ImageJ wound healing size tool to calculate wound closure percentage and rate (Suarez-Arnedo et al., 2020).

### 2.11 Autophagy quantification

Autophagy was assessed using the autophagy detection kit (Abcam^®^, Australia) according to the manufacturer’s instructions. Co-cultures were grown for 24 h in the same manner as described in the cell proliferation assay, except in this case, using phenol-free cell growth media in sterile, dark, clear bottom 96-well microplates. Rapamycin (500 nM) was added at the start of the co-culture as a positive control; 3 h prior to assaying for autophagy, media for cancer cells in negative control wells was replaced with Earle’s balanced salt solution (ThermoFisher Scientific, Australia) with 20 µM chloroquine and re-incubated in humidified 5% CO_2_ at 37 °C. All media was then removed from wells, and the cells were washed twice in 1× assay buffer before staining with the kit’s green detection reagent (2µl/mL) and incubating at 37 °C for 30 min. The stain was then removed. Cells were washed twice with 1× assay buffer to remove excess stain before adding 100 µL of 1× assay buffer to each well and quantifying fluorescence at Ex/Em of 465/534 using the FlexStation 3 plate reader (Molecular Devices, United States).

### 2.12 Apoptosis assay

Cellular apoptosis was evaluated using the Beckman Coulter CytoFLEX Research Flow Cytometer (Beckman Coulter^®^ Life Sciences, Australia) after staining with Annexin V and Propidium iodide (PI), TACS^®^ Annexin V-FITC Apoptosis Detection kit (Trevigen^®^, United States). Co-cultures of cancer cells with bacteria and/or those treated with bacteriophages were incubated for 24 h before replacing the media with fresh media containing 5-fluorouracil or oxaliplatin at a concentration of 100 µM. These were then incubated for a further 24 h in humidified 5% CO_2_ at 37 °C. The cells were then harvested by trypsinisation and washed twice in PBS before staining with PI (2µl/mL) and Annexin V (2µl/mL) in 1× Annexin Binding buffer (ThermoFisher Scientific, Australia). This was incubated in the dark at room temperature for 30 min before capturing 50,000 events by flow cytometry. Data were analysed in the CytExpert software version 2.3.1.22 (Beckman Coulter, Inc., Australia). The proportion of apoptotic and necrotic cells was determined by adding the upper right quadrant (late apoptotic, secondary necrosis), lower left quadrant (early apoptotic – phosphatidylserine exposed, membrane intact) and upper left quadrant (necrotic - membrane compromised, not apoptotic).

### 2.13 Statistical analysis

Experimental data included at least three biological and four technical replicates. All data were imported into and analysed in the IBM^®^ SPSS^®^ software platform version 27 (SPSS, Inc., United States). Normality of quantitative data was determined by the Shapiro-Wilk test with the paired *t-*test being used to compare means between two groups with normally distributed data while the non-parametric counterpart, the Wilcoxon signed-rank test was used for data that significantly deviated from the normal distribution. For two or more groups of data, we compared their means using Analysis of Variance. For all tests, a *p* value of less than 0.05 was considered statistically significant.

## 3 Results

### 3.1 Widespread expression of Gal-GalNAc in human GI cancer cells mediates *F. nucleatum* infection

**Figure 1:**
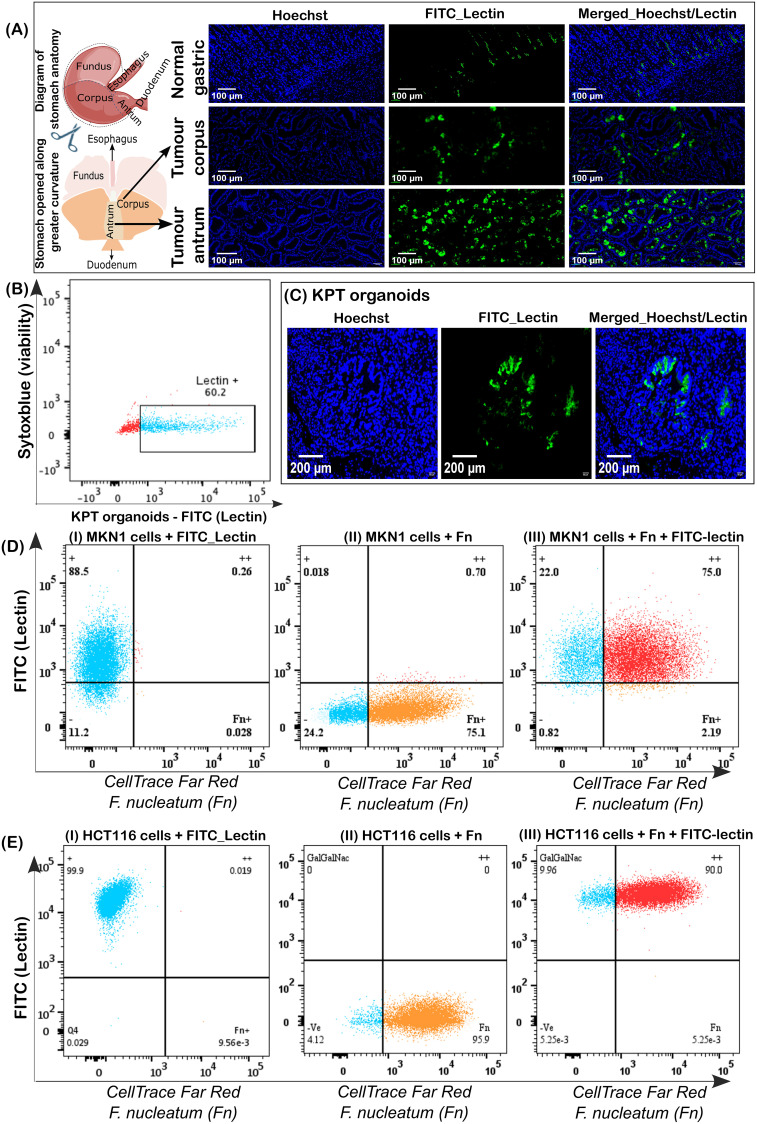
Co-localisation of Gal-GalNAc expression in *F. nucleatum*-infected gastrointestinal tumour cells. **(A)** Visualisation of Lectin bound to gastric corpus and antrum tumour tissues in gp130^F/F^ (FF) mice. **(B)** Gal-GalNAc expression in KPT murine gastric cancer organoids and in C) orthotopic KPT tumours visualised using a confocal microscope. Hoechst fluorescent dye stains chromosomes blue. **(D)** Flow cytometry analysis of Gal-GalNac expression in MKN1 cells infected with cellTrace™ far-red labelled *F. nucleatum*, multiplicity of infection 1:10. **(E)** Flow cytometry analysis of Gal-GalNac expression in HCT116 cells infected with cellTrace™ far-red labelled *F. nucleatum*, multiplicity of infection 1:30 (Fn: *F. nucleatum*).

In gastric tumour tissues (corpus and antrum), we found a significantly higher expression of Gal-GalNAc compared to the wildtype tissues (*p<0.01*) (**Figures 1A, B**) in the *gp130^F/F^* mouse model of inflammatory cytokine-driven intestinal metaplasia and early gastric cancer that affects both antrum and corpus regions of the murine stomach (Tebbutt et al., 2002). In this model, there was a higher expression of Gal-GalNAc in the antrum compared to the corpus tissues, *p < 0.001*. We also observed Gal-GalNAc expression in more than 60% of cells in *Kras^G12D^*, *Pi3kCA^H1047R^* and *Tp53^R172H^* (KPT) mutant-murine cancer organoids (Huber et al., 2024), irrespective of whether the cancer organoids were maintained ex vivo in 3D static cultures or grown as orthotopic tumours *in vivo* (**Figures 1C, D**).

Similarly, we evaluated the level of Gal-GalNac expression in two human GI cancer lines, the gastric adenocarcinoma cell line MKN1 and the colon carcinoma cell line HCT116. Flow cytometry of cells stained with a Gal-GalNAc-specific, FITC-labelled lectin indicated that Gal-GalNAc sugar moieties were displayed on the cell surface of almost every cell (**Figures 1E, F**, left panels). 24 h after infection with fluorescently labelled *F. nucleatum*, indicated an average of 86% of *F. nucleatum* positive cells (represented in **Figures 1D, E**, middle panels) also stained positive for lectin (**Figures 1E, F**, right panels) suggesting that *F. nucleatum* interaction with MKN1 and HCT116 cells is Gal-GalNAc dependent.

### 3.2 Live and fragmented *F. nucleatum* induces proliferation in gastrointestinal cancer cells

**Figure 2:**
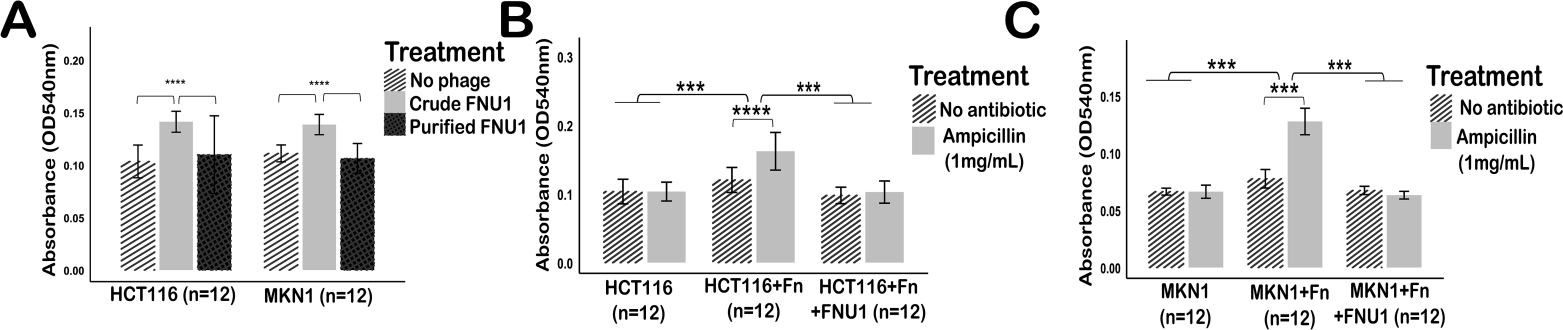
Proliferation of HCT116 and MKN1 cells treated with crude or purified bacteriophage FNU1 preparations **(A)**. Proliferation of HCT116 and MKN1 cells (without bacteriophages or bacteria, in co-cultures with *F. nucleatum*, and exposed to purified FNU1) in the absence or presence of ampicillin **(B, C)**. ****p-value < 0.0001; ***p<0.001.

When a crude preparation of bacteriophage FNU1 (containing approximately 1 × 10^6^ endotoxin units per mL, EU mL) (Binte Mohamed Yakob Adil et al., 2024) was added to the cancer cells, proliferation was significantly increased (**Figure 2A**) (*p* < 0.001). These proliferative effects were negated when FNU1 was purified (to <5 EU mL) using NaCl/PEG precipitation and Triton™ X-100 treatment, removing LPS and other bacterial cellular debris (**Figure 2A**). There was no significant difference between the proliferation of cancer cells (HCT116 or MKN1) in monoculture and those co-cultured with *F. nucleatum* treated with purified bacteriophage FNU1 (*p* = 0.072), with purified bacteriophage alone (*p* = 0.272) or with the antibiotic ampicillin alone (*p* = 0.519). However, ampicillin treatment of the cancer cells (HCT116 or MKN1) when in co-culture with *F. nucleatum* significantly increased proliferation (*p* < 0.001) (**Figures 2B, C**), indicating stimulation of cancer cells by bacterial cell debris. Therefore, apart from the experiments depicted in **Figure 2A**, all other experiments were performed using only purified FNU1 bacteriophage (rid of LPS and other bacterial cellular debris).

### 3.3 FNU1 bacteriophage eliminates intracellular *F. nucleatum*

**Figure 3:**
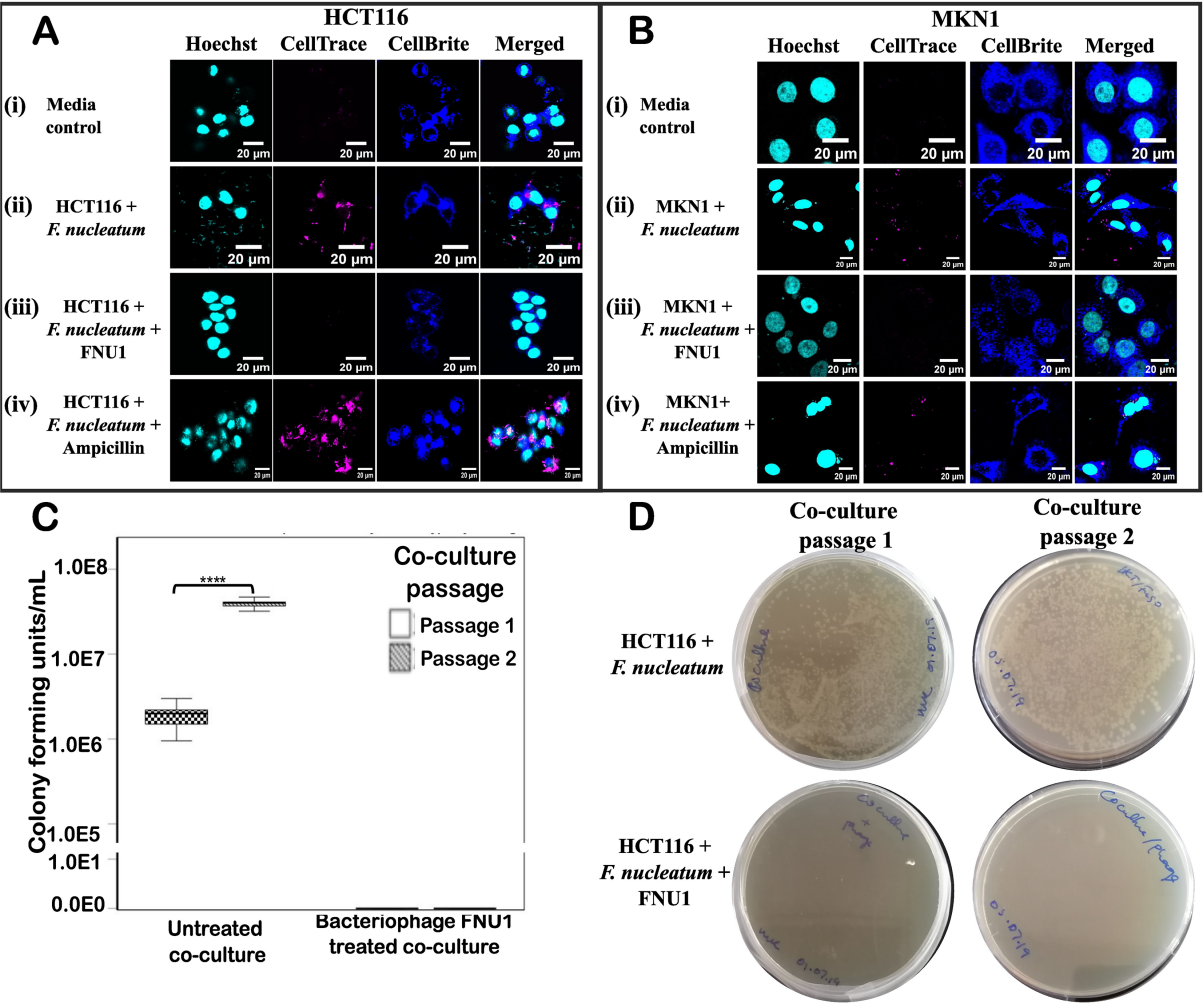
Laser scanning confocal microscopy showing GI cancer cells HCT116 **(A)** and MKN1 **(B)**. The GI cancer cells are marked by blue staining of cytoplasm and cyan staining of nucleus (i); GI cancer cells in co-culture with *F. nucleatum* (magenta) (ii); bacteriophage FNU1 treatment of the co-culture (iii); GI cancer cell/*F. nucleatum* co-culture treated with 1mg/mL of ampicillin (iv). Hoechst post-stain captured the cancer cell nucleus (cyan), CellBrite™ cytoplasm stain (blue), and bacteria pre-stain before culture with CellTrace™ CSFE proliferative dye (magenta). When the co-culture was passaged, viable bacteria in the untreated co-culture were transferred to a petri dish and showed a significant increase (P < 0.001) in the colony counts. Bacteriophage treated co-cultures did not passage any viable bacteria **(C, D)**.

Using laser scanning confocal microscopy, we were able to visualise GI cancer cells (marked by blue staining of cytoplasm and cyan staining of nucleus) (**Figures 3A, B**); when co-cultured with *F. nucleatum* (magenta staining), we observed that *F. nucleatum* cells existed extracellularly and intracellularly (**Figures 3Aii, Bii**). When the co-culture was treated with the bacteriophage FNU1, both intracellular and extracellular *F. nucleatum* were eliminated (**Figures 3Aiii, Biii**). Initially, the minimum inhibitory concentration of the antibiotic ampicillin against *F. nucleatum* using BHI broth dilution was determined at 2 µg/mL. When the co-culture was treated with 1 mg/mL ampicillin the antibiotic was able to eliminate extracellular bacteria but not intracellular *F. nucleatum* (**Figures 3Aiv, Biv**). After passaging we showed that *F. nucleatum* remained viable in the co-culture (**Figures 3C, D**) and following inoculating on BHI agar, observed a significant increase (*p* < 0.001) in the colony counts of *F. nucleatum* between the first and second passage of the co-cultures. No colonies were observed after the first and second passages in bacteriophage treated co-cultures of GI cancer cells (**Figure 3D**).

### 3.4 FNU1 bacteriophage treatment reduces *F. nucleatum-*driven migration of gastrointestinal cancer cells while antibiotic treatment does not

**Figure 4:**
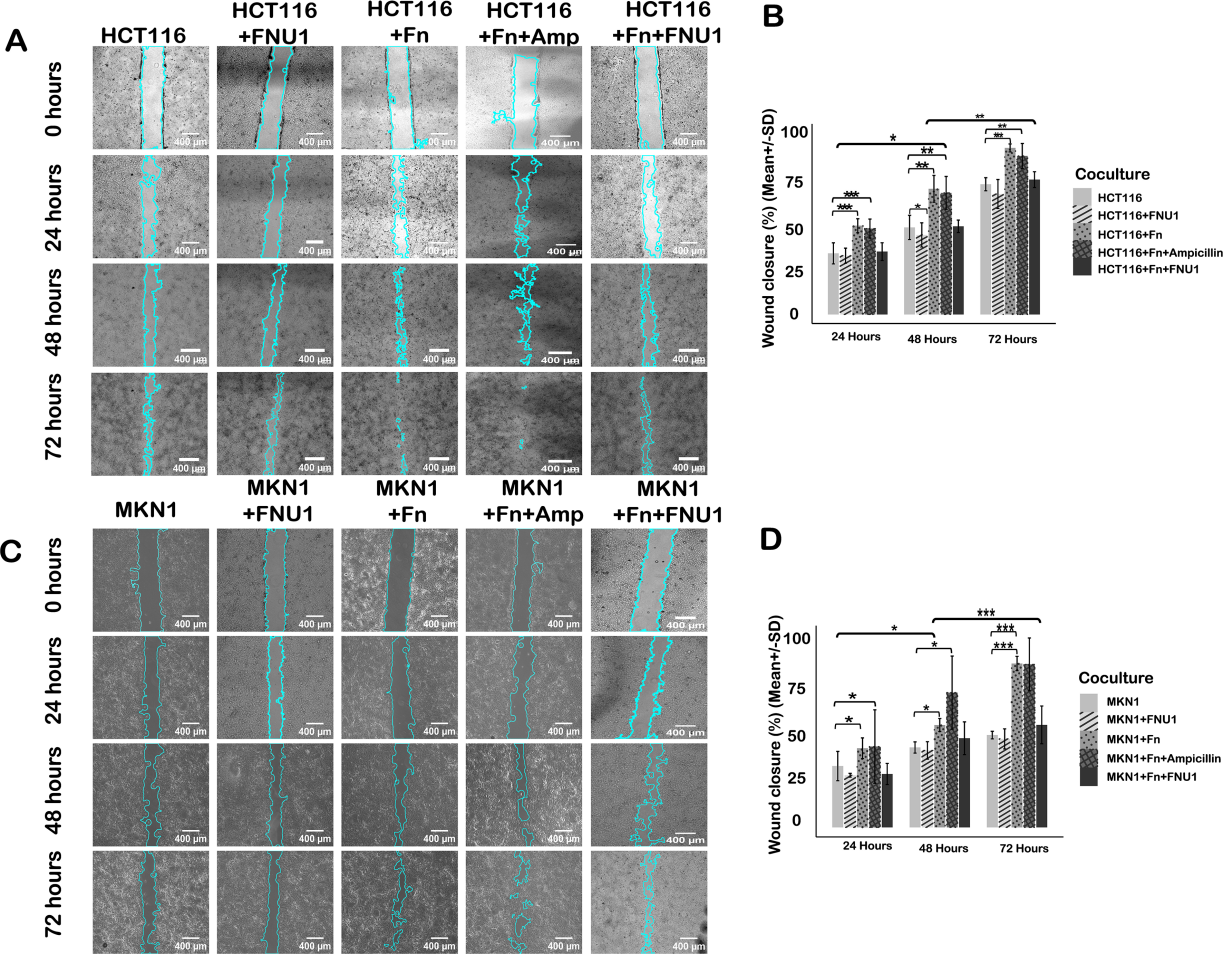
Representative bright field microscope images of wound healing assay for *F. nucleatum* infected gastrointestinal cancer cells treated with FNU1 bacteriophage. Migration of different co-cultures and treatments for HCT116 **(A)** and their proportion of wound closure **(B)**. Migration of different co-cultures and treatments for MKN1 cells **(C)** and their proportion of wound closure **(D)**. *, **, and *** Indicates *p* values of less than 0.05, 0.01, and 0.001, respectively.

Infection of GI cancer cell lines HCT116 and MKN1 with *F. nucleatum* significantly increased wound closure (cell migration) compared to the uninfected control group (**Figure 4**). We assessed the impact of FNU1 bacteriophage treatment on the migratory potential of infected HCT116 and MKN1 cells. In both cell lines, wound closure was significantly lower at 24, 48 and 72 hours (*p* < 0.05, *p* < 0.001 and *p* < 0.01) compared to infected cells. Infected cells treated with FNU1 had similar migration to uninfected cells (**Figure 4**) indicating that FNU1 bacteriophage therapy effectively repressed *F. nucleatum*-driven migration of cancer cells. These findings indicated that FNU1 limited the effects of *F. nucleatum*, thereby attenuating the bacterial-dependent increase in cancer cell migration. The GI cancer cell/*F. nucleatum* cocultures treated with the antibiotic ampicillin did not reduce the migratory effects of *F. nucleatum* and resulted in even higher proliferation of the GI cancer cells than those exerted by co-culturing *F. nucleatum* alone (**Figures 4B, D**), *p* < 0.01.

### 3.5 FNU1 negates *F. nucleatum*-induced autophagy in GI cancer cells

**Figure 5:**
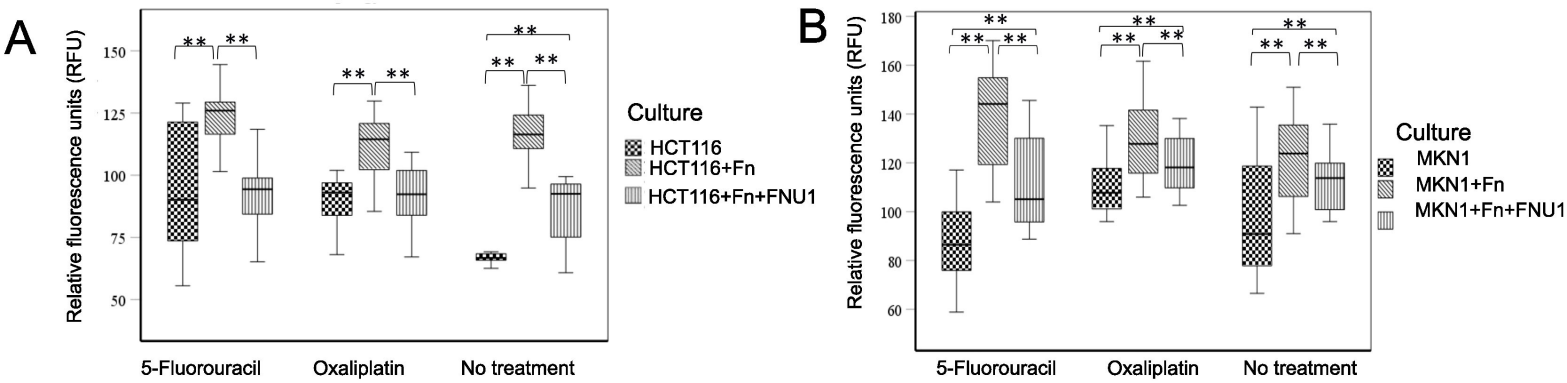
Autophagy flux in **(A)** HCT116 colon cancer and **(B)** MKN1 gastric cancer cells in monoculture, in co-culture with *F. nucleatum*, treated with bacteriophage FNU1 and treated with chemotherapy drugs 5-FU, oxaliplatin and no chemotherapy control. Box plots represent the median value and lower 25th and upper 75th quartiles. **Indicates *p* < 0.01.

*F. nucleatum* increased the autophagic flux in colon cancer cells (**Figure 5A**), (*p* < 0.001) while bacteriophage FNU1 was capable of significantly reducing this effect (*p* < 0.001) but not to control levels *F. nucleatum* increased autophagic flux in both 5-FU (*p* < 0.001) and oxaliplatin (*p* = 0.001) treated co-cultures. In these chemotherapy experiments, FNU1 treatment reduced the autophagic flux to levels similar to uninfected cancer cells (5-FU: *p* = 0.727, oxaliplatin: *p* = 0.267).

As seen in colon cancer cells, the addition of *F. nucleatum* to cultures of MKN1 cells (**Figure 5B**) increased autophagic flux (*p* = 0.002), which was significantly reduced by treating with FNU1 (*p* = 0.005). However, these levels were still higher than those in gastric cancer cells in monoculture (*p* = 0.018). This trend continued when the co-cultures were treated with chemotherapy agents.

### 3.6 Modulation of apoptosis and impact on GI cancer cell proliferation

**Figure 6:**
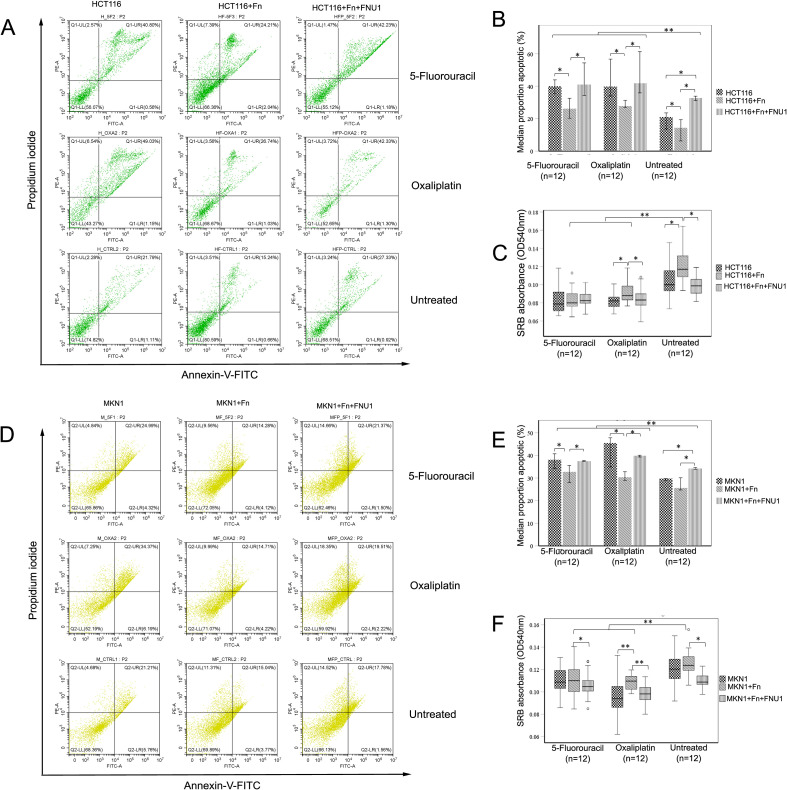
Anti-apoptotic and proliferative effects of *F. nucleatum* in cancer cells treated with chemotherapy drugs 5- fluorouracil and oxaliplatin, and the bacteriophage FNU1. Effects on HCT116 colon cancer cells shown in **(A)** representative flow cytometry scatterplot of propidium iodide and annexin-V, **(B)** summary of flow cytometry data shown as proportion of cells undergoing apoptosis and **(C)** sulforhodamine B assay for cell proliferation. Effects on MKN1 cancer cells are indicated by **(D)** representative flow cytometry scatterplot of propidium iodide and annexin-V, **(E)** summary of flow cytometry data shown as proportion of MKN1 cells undergoing apoptosis and **(F)** sulforhodamine B assay for cell proliferation. Box plots represent the median value and lower 25th and upper 75th quartiles of three biological and four technical replicates. ° indicates an outlier datapoint, * Indicates *p* < 0.05. ** Indicates *p* < 0.01.

Cell death as a result of the co-culture and/or treatment with chemotherapy was calculated as a total proportion of cells stained with annexin V and propidium iodide via flow cytometry for both colon cancer cells (**Figures 6A–C**) and gastric cancer cells (**Figures 6D–F**).

Unlike colon cancer cells, there was no statistically significant difference between the proportion of apoptotic cells in untreated *F. nucleatum*/MKN1 co-cultures and MKN1 cells in monoculture (*p* = 0.113; **Figure 6E**). In the absence of drug treatment, addition of FNU1 significantly increased the proportion of cells in apoptosis, when compared to cancer cells co-cultured with *F. nucleatum* (**Figure 6B**, *p* = 0.028 and **Figure 6E**, *p* = 0.026), or in monoculture (**Figure 6B**, *p* = 0.028 and 6E, *p* = 0.026) in colon and gastric cancer cell lines, respectively.

In colon cancer cells, *F. nucleatum* inhibited apoptosis (*p* = 0.046; **Figure 6B**) and treatment with 5-FU increased apoptosis from a median of 14.5% in untreated *F. nucleatum*/HCT116 co-culture to a median of 26.1%, while in oxaliplatin-treated *F. nucleatum*/HCT116 co-culture, apoptosis was increased to a median of 27.8%. Treatment of HCT116 cells with bacteriophage FNU1 in the absence of chemotherapy increased apoptosis in *F. nucleatum*/HCT116 co-culture to a median of 32.7%. The combination of 5-FU and FNU1 in colon cancer cells co-cultured with *F. nucleatum* increased apoptosis to a median of 41.1%; in similar co-cultures, oxaliplatin with FNU1 increased apoptosis to 41.9%. Therefore, in HCT116/*F. nucleatum* co-cultures, bacteriophage FNU1 increased apoptosis efficacy of 5-FU from 26.1% to 41.1%, while that of oxaliplatin was increased from 27.8% to 41.9%.

In gastric cancer cells (**Figure 6E**), the median apoptosis levels in *F. nucleatum*/MKN1 co-culture were increased from 25.6% to 32.7% with 5-FU and to 30.4% with oxaliplatin. FNU1 increased apoptosis to a median of 34.1% in MKN1/*F. nucleatum* co-cultures. The combination of FNU1 with 5-FU in gastric cancer cell co-culture with *F. nucleatum* further increased apoptosis to 37.4% and FNU1 with oxaliplatin further increased apoptosis to 39.9%.

Interestingly, bacteriophage FNU1 induced greater levels of apoptosis than 5-FU in co-cultures of *F. nucleatum* and colon cancer cells (*p* = 0.015; **Figure 6B**) but not in co-cultures of *F. nucleatum* and gastric cancer cells (*p* = 0.394; **Figure 6E**). Compared to oxaliplatin, bacteriophage FNU1 induced greater levels of apoptosis in both colon and gastric cancer co-cultures (*p* = 0.004; **Figure 6B** and *p* = 0.002; **Figure 6E**, respectively).

### 3.7 FNU1 bacteriophage negates *F. nucleatum*-induced gastrointestinal cancer cell proliferation and resistance to oxaliplatin

Treatment of HCT116 cells with the chemotherapy drugs (5-FU or oxaliplatin) led to a significant reduction in cell viability, irrespective of whether the cells were co-cultured with *F. nucleatum* or not (**Figure 6C**). While *F. nucleatum* infection increased the proliferation of untreated and oxaliplatin-treated HCT116 cells, this effect was not observed after treatment with 5-FU, possibly suggesting that 5-FU exerts greater anti-bacterial activity than oxaliplatin under these culture conditions. Overall, the addition of FNU1 bacteriophage reverted cell proliferation to baseline (**Figure 6C**).

In MKN1 gastric cancer cells, there was no significant difference in cell viability between MKN1 cells in monoculture and those co-cultured with *F. nucleatum* (**Figure 6F**) (*p* = 0.253). However, with the addition of bacteriophage FNU1 to *F. nucleatum*/MKN1 co-cultures resulted in significantly decreased proliferation of gastric cancer cells (*p* = 0.002). With the addition of oxaliplatin, *F. nucleatum* significantly increased MKN1 cell proliferation (*p* < 0.001) while FNU1 treatment resulted in gastric cancer cell proliferation that was significantly decreased (*p* < 0.001). In experiments where 5-FU was added to the culture, *F. nucleatum* did not significantly increase MKN1 cell proliferation compared to those in monoculture (*p* = 0.909) but treatment of *F. nucleatum*/MKN1 co-cultures with FNU1 resulted in a significant reduction in proliferation of the gastric cancer cells (**Figure 6F**) (*p* = 0.012).

## 4 Discussion

The recent recognition of the important role of the microbiome in tumourigenesis has paved the way for its potential manipulation by bacteriophages (Kabwe et al., 2021a). In this study, we have used the bacteriophage FNU1, with selective lytic activity against *F. nucleatum subsp. polymorphum*, to assess efficacy in controlling cancer growth stimulated by this oncobacterium. It has been recognised that *F. nucleatum* binds to Gal-GalNAc on cancer cells through its expression of the Fap2 receptor (Abed et al., 2016). In this study, a higher concentration of Gal-GalNAc was found in the antrum of gastric cancer tissue compared to the corpus, which corelates with data that cancers are more commonly found in the anatomical region of the antrum, than other regions of the stomach (Shang and Pena, 2005). Using co-cultures and antibiotic protection assays, we provide evidence to suggest that *F. nucleatum* uses the Gal-GalNAc receptors for attachment to, and possibly entry into gastric cancer cells, and this protects the bacteria from clearance by ampicillin. *F. nucleatum* increases colon and gastric cancer cell proliferation *in-vitro*, and this oncogenic effect is negated by treatment with FNU1. *F. nucleatum* also promotes the spread or invasiveness of cancer cells, promotes autophagy and inhibits apoptosis in untreated colon and gastric cancer cells, as well as those treated with chemotherapy drugs (Yu et al., 2017; Lu et al., 2019), and we reveal in this study that these effects are also negated/reversed by treatment with FNU1.

The role of *F. nucleatum* in carcinogenesis has been studied, where the presence of this bacterium or its DNA is associated with worse outcomes and treatment failures (Yamamura et al., 2016; Bullman et al., 2017; Kunzmann et al., 2019). The DNA load of *F. nucleatum* in tumour tissues and tumour growth instigated by this bacterium are reduced after treatment with the antibiotic metronidazole in murine models of colorectal (Bullman et al., 2017) and breast cancer (Parhi et al., 2020). Metronidazole is able to penetrate eukaryotic cells and kill intracellular bacteria (Samuelson, 1999). However, its use as an adjunct in cancer therapy may be questionable, as it induces DNA damage, as well as dysbiotic effects on the microbiota, and has been associated with cancer progression (Menéndez et al., 2001; Adil et al., 2018). Similar to our findings, a recent study has used gentamicin to show that *F. nucleatum* enters cancer cells (Kalaora et al., 2021). Ampicillin does not enter eukaryotic cells (Ahrén et al., 2002), and in our study, while allowing the abrogation of extracellular bacteria, not only failed to clear intracellular bacteria but also promoted the overgrowth of the cancer cells in co-culture with *F. nucleatum.* Bacteriophages eliminated intracellular bacteria and kill bacteria more rapidly than antibiotics. it is possible that more LPS is released after antibiotic treatment (Hurley, 1995; Dufour et al., 2017) and coupled to surviving intracellular *F. nucleatum*, antibiotic treatment would promote cancer cell growth. LPS has been shown to have a profound effect on gene expression of colon cancer cells supporting their role in cancer development (Cao, 2023). In our study there was an increase in GI cancer cell proliferation when exposed to crude bacteriophages, i.e., bacteriophage preparations containing host cellular debris including endotoxins. We also showed here that bacteriophage FNU1 is capable of eliminating intracellular *F. nucleatum.* Previously, bacteriophages have been shown to eliminate intracellular bacteria when internalised through endocytosis by phagocytic cells (Kim et al., 2012; Zhang et al., 2017), or carried into the eukaryotic cell by bacteriophage-infected obligate intracellular bacterial cells (Hsia et al., 2000), or traverse non-phagocytic epithelial cells through their receptors (Barr et al., 2013).

To investigate whether *F. nucleatum* reduced the cytotoxicity of 5-FU and oxaliplatin, and if bacteriophage FNU1 was able to negate such effects, we investigated induction of autophagy. Autophagy is a well-characterised mechanism by which *F. nucleatum* induces chemotherapy resistance (Tang et al., 2016; Yu et al., 2017). We demonstrated that FNU1 can significantly reduce the autophagic flux resulting from *F. nucleatum* stimulation of cancer cells in co-culture. We could not find any other study that has investigated bacteriophage capacity to modulate autophagic flux. In addition, our study demonstrated that the chemotherapy resistance induced *by F. nucleatum* through inhibition of apoptosis may be negated by bacteriophage treatment. Previously, bacteriophages against *F. nucleatum* have been used to augment cancer therapy. Although apoptosis was not measured, Zheng and colleagues performed transcriptomic studies showing that elimination of *F. nucleatum* using bacteriophages inhibited the upregulation of anti-apoptotic genes and downregulated pro-autophagy genes (Zheng et al., 2019). In our study we quantified this effect and demonstrated that FNU1 was able to negate oncobacterial-induced chemotherapy resistance.

We demonstrated an increased proliferative effect of *F. nucleatum* in co-cultures with HCT116 colon cancer and MKN1 gastric cancer cells and when these co-cultures were treated with oxaliplatin but not 5-FU. 5-FU is a potent antimicrobial compound through its inhibition of RNA and DNA synthesis and induction of DNA damage in bacterial cells (Zhang et al., 2024). Oxaliplatin on the other hand does not appear to have significant antimicrobial activity in cell culture systems (Gupta et al., 2022). However, it is important to note that these are experimental models, and in a complex tumour microbiome comprising many other bacterial species, the structure and function of cancer drugs such as 5-FU can be altered. For instance, pks+ *Escherichia coli* can modify the chemical structure of 5-FU through converting uracil into 5,6-dihydrouracil, de-activating 5 - FU in the process (LaCourse et al., 2022).

## 5 Conclusion

Our study showed that using bacteriophage FNU1 to control *F. nucleatum* limited the proliferation and migration of cancer cells, and increased their chemosensitivity. Further, FNU1 was capable of reducing autophagic flux induced by *F. nucleatum* which is key to preventing chemotherapy resistance (Yu et al., 2017). These effects led to an increase in the efficacy of chemotherapy in both colon cancer and gastric cancer cells co-cultured with *F. nucleatum*, as measured by the proportion of apoptotic cells. The results suggest that further testing of bacteriophages against oncobacteria using *in vivo* models is warranted in order to manipulate the microbiome and enhance chemotherapeutic efficacy against tumours colonised by these cancer-promoting microbes.

## Data Availability

The original contributions presented in the study are included in the article/supplementary material. Further inquiries can be directed to the corresponding author.
